# Inequalities in health and health risk factors in the Southern African Development Community: evidence from World Health Surveys

**DOI:** 10.1186/s12939-018-0762-8

**Published:** 2018-04-27

**Authors:** Stella M. Umuhoza, John E. Ataguba

**Affiliations:** 1 Department of Health Policy, Economics and Management, School of Public Health, College of Medicine and Health Sciences, University of Rwanda, Kigali, Rwanda; 2 Health Economics Unit, School of Public Health and Family Medicine, Faculty of Health Sciences, University of Cape Town, Anzio Road, Observatory, 7925 South Africa

**Keywords:** Health inequality, Self-assessed health, World Health Survey, Southern African development community

## Abstract

**Background:**

Socioeconomic inequalities in health have been documented in many countries including those in the Southern African Development Community (SADC). However, a comprehensive assessment of health inequalities and inequalities in the distribution of health risk factors is scarce. This study specifically investigates inequalities both in poor self-assessed health (SAH) and in the distribution of selected risk factors of ill-health among the adult populations in six SADC countries.

**Methods:**

Data come from the 2002/04 World Health Survey (WHS) using six SADC countries (Malawi, Mauritius, South Africa, Swaziland, Zambia and Zimbabwe) where the WHS was conducted. Poor SAH is reporting bad or very bad health status. Risk factors such as smoking, heavy drinking, low fruit and vegetable consumption and physical inactivity were considered. Other environmental factors were also considered. Socioeconomic status was assessed using household expenditures. Standardised and normalised concentration indices (CIs) were used to assess socioeconomic inequalities. A positive (negative) concentration index means a pro-rich (pro-poor) distribution where the variable is reported more among the rich (poor).

**Results:**

Generally, a pro-poor socioeconomic inequality exists in poor SAH in the six countries. However, this is only significant for South Africa (CI = − 0.0573; *p* < 0.05), and marginally significant for Zambia (CI = − 0.0341; *P* < 0.1) and Zimbabwe (CI = − 0.0357; *p* < 0.1). Smoking and inadequate fruit and vegetable consumption were significantly concentrated among the poor. Similarly, the use of biomass energy, unimproved water and sanitation were significantly concentrated among the poor. However, inequalities in heavy drinking and physical inactivity are mixed. Overall, a positive relationship exists between inequalities in ill-health and inequalities in risk factors of ill-health.

**Conclusion:**

There is a need for concerted efforts to tackle the significant socioeconomic inequalities in ill-health and health risk factors in the region. Because some of the determinants of ill-health lie outside the health sector, inter-sectoral action is required.

## Background

Internationally, reducing inequalities in health remains a major concern [1, 2]. It is well established, both in developing and developed countries, that (ill)health follows a socioeconomic gradient, to the disadvantage of poorer households, for a large number of diseases and health conditions [1, 3, 4]. This includes child health outcomes [5], healthcare utilisation [6–8], general self-rated health, illness and disability [3, 9–11]. Although there have been observed improvements [12], the same pattern persists over time within countries and the same gradient exists between countries [2, 13, 14]. It is increasingly recognised that social determinants of health (SDH) —as opposed to biological or genetic issues—contribute substantially to this gradient in health [15]. The SDH are circumstances, into which individuals are born, grow up, live, work and age. They include political, economic, social and cultural factors affecting health and well-being of individuals, including education, employment, water and sanitation, housing and infrastructure and social security [15, 16]. Studies indicate that health disparities caused by social and economic determinants are deemed unfair and avoidable because they are produced by circumstances that can be addressed through policies [16, 17]. This is also the case with many health risk behaviours (e.g. smoking, alcohol abuse, physical inactivity and unhealthy diet) that are found to exert a strong influence on health [15] and are disproportionately distributed among individuals of low socioeconomic status (SES). They perpetuate the burden of ill-health and poverty within this group [18, 19].

In Africa including in the Southern African Development Community (SADC) region, few studies have assessed socioeconomic inequalities in a number of health-related outcomes and selected disease conditions [3, 20–22]. The findings from these studies also confirm the existence of the socioeconomic gradient in ill-health. However, there is a dearth of studies that broadly and comprehensively examine socioeconomic inequalities in health, health risk factors and the wider determinants of health. Only recently, a study on the social determinants of ill-health in South Africa demonstrated the importance of other sectors including social protection, employment and the provision of basic social services (knowledge and education, housing and infrastructures) in tackling disparities in health in the country [17].

Globally, with the exception of south-east Asia, sub-Saharan Africa contributes substantially to the global burden of disease [23]. In sub-Saharan Africa, the contribution of the SADC region is significant due to the burden of HIV/AIDS [24]. Sub-Saharan Africa’s contribution to other health risk factors remains substantial especially the use of alcohol among African men [23]. In addition to addressing the burden and socioeconomic distribution of ill-health and diseases, it is recognised that an understanding of the role of these health “risk factors is important for developing clear and effective strategies for improving global health” ([25] p.v). Thus, given the overall contribution of the SADC region to the burden of disease in sub-Saharan Africa, this paper specifically investigates socioeconomic inequalities in health and health risk factors in the SADC region. It tries to investigate, together, inequality in poor self-assessed health (SAH) and the distribution of risk factors of ill-health among the adult population in the six SADC countries (Malawi, Mauritius, South Africa, Swaziland, Zambia and Zimbabwe) where comparable data exist. This evidence is invaluable to policy makers to develop targeted and effective policy initiatives that address the unequal distribution of health and the SDH.

The rest of this paper is structured as follows. The next section provides a brief profile of the selected study countries and the methodology section follows. Next, the results from the analysis are given and the subsequent section discusses the results with some policy implications. The last section provides a brief conclusion to the study.

## A brief overview of the study countries

The SADC was established in 1980. Currently, it comprises 15 member states (Angola, Botswana, Democratic Republic of Congo, Lesotho, Madagascar, Malawi, Mauritius, Mozambique, Namibia, Seychelles, South Africa, Swaziland, Tanzania, Zambia and Zimbabwe). However, in this paper, as will be discussed in the methods section, only the countries with available data are included in our analysis. The SADC is a regional integration group which aims to “reduce economic dependence particularly, but not only, on South Africa; to forge links to create genuine and equitable regional integration; to mobilize resources for implementing national and interstate policies; and to take concerted action to secure international co-operation within the framework of the strategy of economic liberation” [26]. Table 1 provides an overview of key indicators for the six study countries. Briefly, population size varies from 1.3 million people in Mauritius to 54.8 million in South Africa. The population growth rate ranges from 0.18% in Mauritius to 3.07% in Malawi and Zambia. Countries with moderate population growth rates include Swaziland and South Africa with growth rates estimated at 1.47% and 1.58%, respectively. These countries are at different levels of economic development. Mauritius and South Africa are upper-middle-income countries while Malawi and Zimbabwe are low-income countries. Average per capita gross domestic product (GDP) ranged from $272 in Malawi to $7,166 in Mauritius. GDP growth rate in 2014 was highest in Zambia (6.0%) and lowest in South Africa (1.5%). Overall, the annual GDP growth rate in the SADC region averaged 4.7% between 2003 and 2013. Generally, the service sector contributes significantly to GDP growth given the expanding tourism industry in these countries [27]. In comparison to the other countries, South Africa is the most urbanised (64.3%).

**Table 1:** Selected indicators for the study countries

Description	Malawi	Mauritius	South Africa	Swaziland	Zambia	Zimbabwe	Source
Population							
Populations (2015, million)	17.22	1.265	54.77	1.287	16.21	15.60	[31]
Population growth rate (2014, annual, %)	3.07	0.18	1.58	1.47	3.07	2.31	[31]
Urban population (2014, % of total)	16.10	39.81	64.30	21.32	40.47	32.5	[31]
Economy and labour market							
Country type (income level)	LIC	UMIC	UMIC	LMIC	LMIC	LIC	[31]
GNI per capital (2014, PPP, US$)	780	18,290	12,700	5,940	3,860	1,710	[31]
GDP per capita (Constant 2005 US$, 2014)	272	7,116	6,086	2,522	1,081	475	[31]
GDP growth rate (% per annum; 2014)	5.7	3.6	1.5	2.5	6.0	3.9	[31]
Unemployment rate (2014, % of total labour force)	7.5	7.7	25.1	22.3	13.3	5.4	[31]
Gini index (year)	0.439 (2013)	0.358 (2012)	0.634 (2011)	0.515 (2013)	0.575 (2013)	0. 501 (2006)	[31]
Human development							
Human Development Index (HDI) (2014)	0.445	0.777	0.666	0.531	0.586	0.509	[28]
HDI rank (2014)	173	63	116	150	139	155	[28]
Inequality-adjusted HDI (2014)	0.299	0.666	0.428	0.354	0.384	0.372	[28]
Gender Inequality index (GII) (2014)	0.611	0.419	0.407	0.557	0.587	0.504	[28]
GII rank (2014)	140	88	83	128	132	112	[28]
Environment determinants: housing and infrastructure							
Access to improved water source (2015, % of population)	90.2	99.9	93.2	74.1	65.4	76.9	[30]
Access to improved sanitation facilities (2015, % of population)	41.0	93.1	66.4	57.5	43.9	36.8	[30]
CO_2_ emissions (2012, million metric tons per capita)	0.1	3.1	9.3	0.9	0.2	0.7	[31]
Electrification rates (2012, % of the population)	9	100	85	27	26	40	[91]
Health status							
Maternal mortality ratio (2015, per 100,000 live births)	634	53	138	389	224	443	[29]
Under-5 mortality rate (2015, per 1000 live births)	64.0	13.5	40.5	60.7	64.0	70.7	[29]
Life expectancy at birth (2015, in years)	58.3	74.6	62.9	58.9	61.8	60.7	[29]
HIV/ AIDS (2015, adults aged 15 to 49 prevalence rates)	9.1	0.9	19.2	28.8	12.9	14.7	[24], (http://www.unaids.org/en/regionscountries/countries)
Health financing							
Total expenditure on health (THE) as % of GDP (2014)	8.3	4.8	8.9	8.4	5.0	n/a	[29]
Public health expenditure as % of GDP (2011)	6.2	2.4	4.31	5.6	3.7	n/a	[29]
GGHE as % of total expenditure on health (2014)	52.7	49.2	48.2	75.7	55.3	n/a	[29]
GGHE as % of total government expenditure (2012)	16.2	9.5	14.0	18.1	12.6	n/a	[29]
Per capital total expenditure on health (PPP int. $; 2014)	93	896	1 148	587	195	n/a	[29]

Notes:

*LIC* low-income countries, *LMIC* low-middle income country, *UMIC* upper-middle-income country;

*GNI* gross national Income, *THE* total expenditure on health, *GDP* gross domestic product, *GGHE* General Government Expenditure on health, *CO*_*2*_ carbon dioxide, *n/a* not available, *WHO* World Health organization, *IEA* International Energy Agency, *UNDP* United Nations Development Programme, *UNAIDS* the Joint United Nations Program on HIV

Mauritius’ Human Development Index estimated at 0.77 (ranked 66th globally) is the highest in Africa [28]. In terms of health, life expectancy is significantly higher in Mauritius and South Africa but lowest in Malawi [29]. Even though most countries made substantial progress towards the Millennium Development Goals (MDGs) target for drinking water and sanitation infrastructure, more than half of populations in Zimbabwe, Malawi and Zambia do not have access to improved sanitation facility [30]. In comparison to other countries, Mauritius is exceptional in having made remarkable progress in sanitation infrastructure (93.1%) and attained universal access to improved water source, electricity and clean household fuel.

The World Bank’s indicators show that South Africa, with Gini index estimated at 0.63, ranks as third most unequal country in the SADC region after Namibia (0.66) and Seychelles (0.66). The Gini index is a measure of income inequality that ranges between zero and one. The closer its value is to one, the more unequal are incomes in the country. Zambia (0.58) and Swaziland (0.52) also present with high unequal income distribution [31]. In addition, most of the study countries suffer from high unemployment and high HIV prevalence, which exacerbates ill-health and health disparities within and between countries. In Swaziland, for example, about 28% of people living with HIV were aged 15–49 years, compared to 0.9% in Mauritius (http://www.unaids.org/en/regionscountries/countries). Although communicable diseases still predominate in these countries, evidence also points to the growing burden of non-communicable diseases (NCDs), contributing to a rising “double-burden of disease” [32].

In relation to health financing, total health expenditure as a share of the country’s GDP ranges from 8.9% in South Africa to 5.0% in Zimbabwe. Mauritius (2.4%) and Zambia (3.7) have the lowest levels of government spending on health as a proportion of GDP. Total government expenditure allocated to health in Zambia (18.1%) and in Malawi (16.2%) exceeded the Abuja target of 15% [33]. However, this is not the case for the other countries including Mauritius and South Africa.

## Methods

### Data

Data come from the 2002/04 World Health Survey (WHS) conducted by the World Health Organization (WHO). The WHS was conducted in 70 countries across all the six WHO regions to provide a valid, reliable and internationally comparable source of population data on the health status of adults aged 18 years or older. The WHS is a cross-sectional household survey that uses a multi-stage cluster design; the geographical clusters were utilised as primary sampling units (PSUs). These PSUs were non-overlapping and are used as grouping or clustering variables in this analysis [34]. All samples were probabilistically selected with every individual being assigned a non-zero probability of being selected.

Household and individual level questionnaires were used to collect data about basic socio-demographic characteristics, household expenditure and assets, health-related outcomes, healthcare utilisation, risky health behaviours and environmental factors. Within each household, a knowledgeable adult member (aged 18 years or older) was randomly selected using the Kish table method to complete an interview [35]. Specifically, data from Malawi, Mauritius, South Africa, Swaziland, Zambia and Zimbabwe were used in this analysis. These are the countries within the SADC region where the WHS data are available. The final datasets contained data on 5551 households for Malawi, 3968 households for Mauritius, 2629 households for South Africa, 3070 households for Swaziland, 4165 households for Zambia and 4264 households for Zimbabwe. In all the countries, sampling weights were included and adjusted for to account for post-stratification corrections and for non-response [34].

### Self-assessed health

SAH is an indicator of health status and has been extensively used in international comparisons and assessment of inequality in health [2, 10, 13]. Although SAH is subjective and self-reported and prone to possible reporting bias [36], it is strongly linked to several health outcomes including subsequent utilisation of medical care [37], functional ability [38], mortality [39] and morbidity [40]. SAH was obtained from respondents’ assessment of their current health status on a five-point scale (very well, good, moderate, bad, or very bad). As in many previous studies [2, 13, 17], SAH was further dichotomised to “poor health” = 1 (combining bad and very bad). “Good health” comprises the remaining categories. A reliability test was carried out to ascertain consistency in the dichotomisation [41].

### Social determinants of health variables

Apart from SAH, other variables (i.e. the SDH) were included in the analysis to assess inequalities in their distribution (Table 2). Four modifiable NCD risk factors available in the WHS data were considered in this paper; current daily smoking, heavy episodic alcohol consumption, low fruit and vegetable consumption, and physical inactivity. These risk factors are most often examined in the literature [18, 42]. Environmental health risk factors include source of drinking water, type of sanitation facility and type of cooking fuels (energy). The environmental determinants have a significant impact on child survival and general well-being in less developed countries [43, 44]**.**

**Table 2:** Summary description of NCDs risk factors and environmental determinants of health

Risk factors and SDH	Description	Categorisation
NCDs risk factors		
Currently smoking	Self-reported use of any kind of tobacco product, including cigarettes, cigars, or pipes, either daily or occasionally	1 = adults that smoke daily or occasionally within the week 0 = adults that do not smoke at all^a^
Heavy episodic alcohol drinking	Self-reported consumption of at least 4 (for women) or 5 (for men) standard alcoholic drinks on a single drinking occasion on at least 1 day of the preceding week.	1 = consuming at least 4 (for women) or 5 (for men) standard alcoholic drinks on a single occasion on at least 1 day of the previous week^b^ 0 = otherwise
Inadequate fruit and vegetable consumption	Self-reported consumption of less than 5 total servings of fruit and vegetable per day (a consumption of less than 400 g per day)	1 = adults consuming less than 5 total servings of fruit and vegetable per day^c^ 0 = otherwise
Physical inactivity	Self-reported physical activity of less than: (i) 150 min of moderate–intensity activity per week; (ii) 75 min of vigorous–intensity activity per week; (iii) the recommended minimum of at least 600 metabolic equivalents-minutes (MET– minutes) per week	1 = an adult that does not meet any of the minimums in (i), (ii) or (iii) as described for self-reported physical activity^d^ 0 = otherwise
Environmental determinants of health		
Unimproved drinking-water sources	Self-reported use of any unimproved sources of drinking-water such as an unprotected spring or dug well, a cart with small tank/drum, tanker truck and surface water (river, dam, lake, stream, canal, irrigation channels)	1 = adult in a household that uses an unimproved source of drinking water^e^ 0 = otherwise
Unimproved sanitation	Self-reported use of any unimproved sanitation facilities, including flush or pour-flush to elsewhere, pit latrine with slab or open pit, bucket, hanging toilet or hanging latrine and no facilities or bush or field (open defecation)	1 = adult in a household that uses an unimproved sanitation facility^e^ 0 = otherwise
Unclean cooking source	Self-reported use of any unclean fuel for cooking (non-biomass fuels) ranging from coal, charcoal, wood, crop residues or dung	1 = adult in a household that uses an unclean cooking source^f^/ biomass fuel 0 = otherwise

Notes:

^a^ This is based on Hosseinpoor AR, Bergen N, Kunst A, Harper S, Guthold R, Rekve D, d’Espaignet ET, Naidoo N and Chatterji S [18], Moradi G, Mohammad K, Majdzadeh R, Ardakani HM and Naieni KH [61]; ^b^ This is based on Hosseinpoor AR, Bergen N, Kunst A, Harper S, Guthold R, Rekve D, d’Espaignet ET, Naidoo N and Chatterji S [18], World Health Organization [92]; ^c^ This is defined based on Hosseinpoor AR, Bergen N, Kunst A, Harper S, Guthold R, Rekve D, d’Espaignet ET, Naidoo N and Chatterji S [18], Peltzer K and Phaswana-Mafuya N [75], Amine E, Baba N, Belhadj M, Deurenbery-Yap M, Djazayery A, Forrester T, Galuska D, Herman S, James W, M’Buyamba J, Katan M, Key T, Kumanyika S, Mann J, Moynihan P, Musaiger A, Prentice A, Reddy K, Schatzkin A, Seidell J, Simpopoulos A, Srianujata S, Steyn N, Swinburn B, Uauy R, Wahlqvist, M., Zhao-su W and Yoshiike N [93]; ^d^ This is based on Hosseinpoor AR, Bergen N, Kunst A, Harper S, Guthold R, Rekve D, d’Espaignet ET, Naidoo N and Chatterji S [18], Organization WH [94]; ^e^ This is based on World Health Organization and UNICEF [30]; ^f^ This is based on Ataguba JE-O, Day C and McIntyre D [17], Williams KN, Northcross AL and Graham JP [95]

### Constructing a measure of socioeconomic status

SES is assessed in this paper using household expenditure. This approach is similar to other studies using the WHS [45]. This comprises expenditures on both frequently purchased and non-frequently purchased goods and services. All household expenditures were annualised. In general, some household factors exert their influence on household expenditure pattern. Examples include the number of individuals within a household, composition of the household members by sex, age, marital status and number of children [46, 47]. In addition, some goods and services consumed by households have a “public good” characteristic, meaning they yield benefits for the entire household not just the primary consumer. These shared goods within the household are the root cause of economies of scale [47, 48]. To account for these, and for any meaningful household level analysis, household expenditures were adjusted for household size and composition [47] to obtain per adult equivalent expenditure.

An adult equivalent household size (*E*) can be conveniently defined as:

1 $$ E={\left(A+\alpha K\right)}^{\beta } $$

where *A* is the number of adults (18 years and above) in the household, *K* is the number of children (under 18 years), *α* is the measure of the weight (adjustment factor) accorded to children relative to adults [46] and *β* is the elasticity capturing economies of scale. The values of *α* and *β* are set at 0.5 and 0.75 respectively. Although the choice of values for *α* and *β* is subjective, this analysis uses these values as applied in recent studies in Africa [6, 17, 47]. Total expenditure is divided by the estimate of *E* for each household to obtain per adult equivalent household expenditure (i.e. the equivalent of per capita household expenditure).

### Analytical method for assessing socioeconomic inequality in health and health risk factors

In the literature, various approaches are used to quantify health inequalities ranging from relatively simple to more sophisticated measures —the range, Gini coefficient, Pseudo-Gini coefficient, index of dissimilarity, slope index (and relative index) of inequality and the concentration index [49, 50]. Any good summary measure of socioeconomic inequality in health has to satisfy three minimum conditions: (i) reflect the socioeconomic dimensions of health, (ii) reflect the experiences of entire population distribution rather than the top and bottom SES groups, and (iii) be sensitive to the changes in the distribution and size of population across socioeconomic groups [50]. Only the slope index of inequality and the concentration index fulfil these properties.

This paper therefore used the concentration index (CI) [49, 50] to assess socioeconomic inequality in the distribution of poor SAH and the risk factors of ill-health (see Table 2) in the selected SADC countries. The CI has been extensively used in the health inequality literature including for multi-country analysis [2, 3, 12, 20, 51, 52]. The CI is derived from the concentration curve (CC), which is a plot of the cumulative proportions of the health variable (e.g. poor SAH or a health risk factor) on the vertical axis against the cumulative proportion of the population, ranked by SES on the horizontal axis. The CI corresponds to twice the area between the concentration curve and the line of equality (i.e., a 45-degree diagonal line). The CI ranges between − 1 and + 1, with negative (positive) values corresponding to “pro-poor” (“pro-rich”) inequality. The larger the absolute value of the CI, the wider the inequalities in the distribution of poor SAH or health risk factor [49, 50].

Given that the CCs may cross each other, a statistical “dominance-test” is performed, especially when countries are compared via concentration curves or indices [50]**.** Dominance tests are useful to statistically assess if inequalities are pro-poor or pro-rich along the entire distribution of per adult equivalent expenditure. For instance, inequality in poor SAH in Country A is dominated by that in Country B if the concentration curve of poor SAH in Country B lies everywhere above the corresponding concentration curve for Country A. This paper uses the multiple comparison approach (MCA) to test if significant differences exist between curves at 19 quantile points [48, 53].

In this paper, the CI is estimated using the “convenient regression approach” [49]:

2 $$ 2{\sigma}_r^2\left(\frac{y_i}{\mu_y}\right)=\alpha +\beta {r}_i+{\varepsilon}_i $$

where *β* is the estimated unstandardised concentration index, *y*_*i*_ is the level of the dichotomous poor SAH (or health risk factor) variable for individual *i*, ${\sigma}_r^2$ is the variance of the fractional rank of SES (*r*), *μ*_*y*_ is the mean of health variable (or health risk factor variable), and *ε*_*i*_ is the stochastic error term.

The concentration index *β* is further standardised to remove the confounding influences of demographic variables and establish a ‘refined’ association between the ill-health variable and SES. This is because age and sex are correlated with either health status or SES, or both [48].

This paper uses the indirect standardisation method to standardise the *y*-variable to obtain ${\widehat{y}}_i^{is}$, which is used to compute the indirectly standardised CI using eq. (2). The indirectly standardized *y*-variable $\left({\widehat{y}}_i^{is}\right)$ is obtained as:

3 $$ {\widehat{y}}_i^{is}={y}_i-{\widehat{y}}_i^x+\overline{y} $$

where $\overline{y}$ is the sample mean of *y* and ${\widehat{y}}_i^x$ is the predicted *y*-variable in a linear regression using the confounding *x*-variables (age and sex) as predictors

4 $$ {y}_i=\alpha +{\sum}_j{\beta}_j{x}_{ji}+{\varepsilon}_i $$

The indirectly standardised *y*-variable $\left({\widehat{y}}_i^{is}\right)$ is equivalent to the distribution of the *y*-variable (poor SAH and risk factor of ill-health) that would be expected regardless of the distribution of age and sex across household expenditure.

For bounded variables, the concentration index depends upon *μ*_*y*_ implying that comparison across populations with different mean levels is not suitable [54]. For large samples in the case of dichotomous variables, the lower and upper bounds would become *μ*_*y*_ − 1 and 1 − *μ*_*y*_ respectively [55]. Wagstaff’s [55] suggestion is to normalise the concentration index by (1 − *μ*_*y*_). However, Erreygers [56] notes that Wagstaff’s [55] ad hoc normalisation will “blow up the levels of measured inequality for distributions with either high or low means” ([56] p.523) as opposed to that proposed in Erreygers [54]. Mathematically, in the case of a binary variable, Erreygers index is a weighted function of that proposed by Wagstaff [3].

Thus, this paper uses the Erreygers corrected index (*E*_*C*_) to normalize the concentration index [54]. The index (*E*_*C*_) allows for comparison of groups of people that could present different levels of average health and it can be computed as [57]:

5 $$ {E}_C=\left(4{\mu}_y/b-a\right) CI $$

where *CI* is the standardised concentration index, *a* and *b* are respectively, the upper and lower bounds of the health variable. The Erreygers corrected index is interpreted similarly as CI.

All statistical analyses were performed in Stata® version 14 [58] after accounting for clustering and unequal probability for the WHS data. The Human Research Ethics Committee (HREC) of the Faculty of Health Sciences, University of Cape Town, approved this research.

## Results

### Descriptive statistics

The female population is greater than 50% in all the countries (Table 3). This is more so in Zimbabwe (> 60%). The average age of adults was above 35 years in the six SADC countries. Poor SAH varied between countries with the lowest proportion in Malawi (22.5%) and the highest proportion in Swaziland (67.1%). Smoking prevalence varied from nearly one-tenth in Swaziland to one quarter in South Africa. On average, except for South Africa, the prevalence of heavy episodic drinking among adults was less than 10%. Inadequate fruit and vegetable intake was very common in all the countries. The lowest consumption level was found in Malawi (39.8%) and the highest in Mauritius (89.3%). The prevalence of physical inactivity was considerably higher (> 40%) among adults in Swaziland and South Africa but lower than 30% in the other countries. Access to improved drinking-water source, improved sanitation and clean cooking source was consistently higher among households in the richer countries (Mauritius and South Africa). Households in Malawi (98.5%), Zambia (87.2%), Swaziland (67.9%) and Zimbabwe (66.2%) depended heavily on unclean cooking fuel such as charcoal, wood and agricultural residues.

**Table 3:** Descriptive statistics

Variables	Malawi	Mauritius	South Africa	Swaziland	Zambia	Zimbabwe
Socio-demographic						
Mean age in years (standard deviation)	35.30 (16.20)	41.18 (15.56)	37.57 (14.47)	38.87 (16.77)	35.40 (14.99)	37.04 (10.07)
Female (%)	56.02	50.43	52.40	52.96	52.74	60.13
Poor SAH (%)	22.36	34.26	27.69	67.10	27.31	44.47
NCDs risk factors						
Currently smoking (%)	14.60	22.62	25.37	9.19	14.14	12.82
Heavy episodic alcohol drinking (%)	2.80	4.10	11.44	1.83	7.37	4.59
Inadequate fruit and vegetable intake (%)	39.83	89.27	69.44	76.04	77.70	86.26
Physical inactivity (%)	15.01	26.38	49.95	41.15	23.34	17.80
Environmental determinants of health						
Unimproved drinking-water source (%)	20.85	0.00^a^	6.07	32.08	39.23	17.53
Unimproved sanitation toilet (%)	20.12	0.09	11.18	20.13	26.92	27.51
Unclean cooking source /Biomass fuel (%)	98.45	1.33	20.92	67.90	87.20	66.15

^a^Mauritius records 100% of the households with access to improved drinking-water sources

### Socioeconomic inequalities in poor SAH and health risk factors

The Erreygers corrected concentration indices for poor SAH were negative in most countries (Table 4) except in Mauritius (*E*_*C*_= 0.0026) where it is pro-rich. However, this pro-rich inequality in poor SAH is not statistically significant. Statistically significant ‘pro-poor’ inequalities in poor SAH were observed for South Africa (*E*_*C*_= − 0.0573; *p* < 0.05), marginally for Zimbabwe (*E*_*C*_= − 0.0357; *p* < 0.10) and marginally for Zambia (*E*_*C*_= − 0.0341, p < 0.10). The pro-poor inequality indicates that poor health was generally more concentrated amongst the poor than among the rich.

**Table 4:** Socioeconomic inequalities in self-assessed health and social determinants of health, by country 2002–04

	Malawi	Mauritius	South Africa	Swaziland	Zambia	Zimbabwe
Overall health						
Poor SAH	−0.0150 (0.0206)	0.0026 (0.0177)	−0.0573** (0.0234)	− 0.0250 (0.0318)	− 0.0341* (0.0182)	− 0.0357* (0.0211)
NCDs risk factors						
Smoking	−0.0704*** (0.0155)	− 0.0404*** (0.0155)	− 0.0206 (0.0206)	−0.0008 (0.0265)	− 0.0190 (0.0142)	−0.0168 (0.0170)
Heavy episodic drinking	0.0197*** (0.0043)	−0.0115 (0.0076)	−0.0124 (0.1443)	− 0.0023 (0.0086)	0.0583*** (0.0121)	0.01622 (0.0101)
Inadequate FV intake	−0.0548** (0.0232)	−0.0554*** (0.0128)	− 0.0723*** (0.0269)	−0.2205*** (0.0303)	− 0.0808*** (0.0189)	−0.0167 (0.0165)
Lower level of PA	0.0020 (0.0156)	−0.0033 (0.0181)	0.0047 (0.0278)	0.0313 (0.0332)	−0.0826*** (0.0253)	0.0457** (0.0192)
Environmental determinants of health						
Unimproved drinking- water source	−0.1654*** (0.0197)	N/A	−0.0325*** (0.0110)	−0.1649*** (0.0394)	− 0.3053*** (0.0248)	−0.1293*** (0.0153)
Unimproved sanitation/toilet facility	−0.1352*** (0.0160)	−0.0016*** (0.0006)	− 0.0589*** (0.0146)	−0.1735*** (0.0279)	− 0.3360*** (0.0172)	−0.2719*** (0.0160)
Unclean cooking source (biomass fuel)	−0.0521*** (0.0066)	−0.0252*** (0.0052)	− 0.1039*** (0.0202)	−0.3627*** (0.0357)	− 0.3203*** (0.0173)	−0.4297*** (0.0171)

*Notes*: *p < 0.10, **p < 0.05, ***p < 0.01

Erreygers corrected concentration indices (*E*_*c*_) with standard error in parenthesis

*FV* fruit and vegetable, *PA* physical activity, *N/A* not applicable because 100% of participants had access to an improved drinking water source

The inequalities in poor SAH can be compared between countries through statistical dominance tests on the associated concentration curves. This is done by using the pairwise comparisons shown in Table 5. The concentration curves of poor SAH for Malawi, Zambia and Zimbabwe dominate the concentration curve for Mauritius. This indicates that the concentration curve of poor SAH for Mauritius lies below the concentration curves for the other countries. The concentration curves of poor SAH for South Africa, Zambia and Zimbabwe dominate that for Swaziland. While the concentration curve of poor SAH for Malawi dominated that of South Africa, the other pairwise comparisons of concentration curves suggest non-dominance, indicating that the concentration curves either cross each other or were not statistically different from each other.

**Table 5:** Results of dominance for poor self-assessment of health

Countries	Mauritius	South Africa	Swaziland	Zambia	Zimbabwe
Malawi	RDC	RDC	ND	ND	ND
Mauritius		ND	ND	CDR	CDR
South Africa			RDC	ND	ND
Swaziland				CDR	CDR
Zambia					ND

Notes:

*RDC* - concentration curve of row dominates that of column

*CDR* - concentration curve of column dominates that of row

*ND* - non-dominance or curves crossing

### Socioeconomic inequality in NCD risk factors

The selected NCDs risk factors are generally more concentrated among the poor than among the rich (Table 4). In all countries, the Erregyers corrected concentration indices for smoking and low levels of fruit and vegetable consumption were negative, showing that poorer individuals were more likely to smoke and less likely to eat the recommended daily serving of fruit and vegetables than their richer counterparts. However, the level/magnitude differed between countries. Although tobacco consumption was concentrated among the poor in all countries, it was only statistically significant for Malawi (*E*_*c*_ = − 0.0704; *p* < 0.01) and Mauritius (*E*_*c*_ = − 0.0404; *p* < 0.01). Also, fruit and vegetable consumption was statistically significant for all countries except for Zimbabwe (*E*_*c*_ = − 0.0167; *p* > 0.1). Heavy episodic drinking was significantly pro-rich in Malawi (*E*_*c*_ = 0.0197; *p* < 0.01) and Zambia (*E*_*c*_ = 0.0583; *p* < 0.01), implying that richer individuals were more likely to be heavy drinkers in these countries. However, a pro-poor pattern was observed for the other countries, but this pattern was not statistically significant. Socioeconomic inequality in physical inactivity was also mixed. Pro-rich socioeconomic inequality was observed in four of the six countries (Malawi, South Africa, Swaziland and Zimbabwe) but this was only statistically significant for Zimbabwe (*E*_*c*_ = 0.0457, *p* < 0.05). While a pro-poor inequality was observed for Mauritius and Zambia as poorer adults were less likely to engage in physical exercise. However, this was only statistically significant for Zambia (*E*_*c*_ = − 0.0826; *p* < 0.01).

**Fig. 1:**
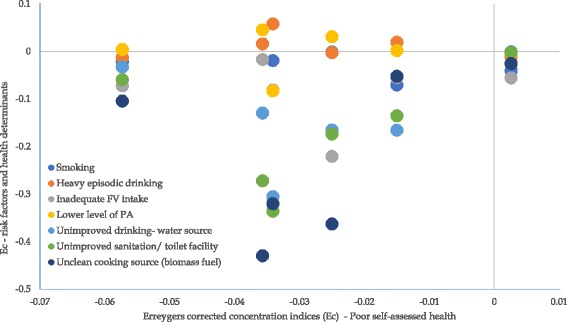
Correlation between inequality in poor self-assessment of health and inequalities in the social determinants of health

The concentration indices for poor SAH and those for the selected SDH and NCD risk factors plotted in Fig. 1 show that inequalities in poor SAH tend to be positively related to inequalities in the SDH.

### Socioeconomic inequality in environmental determinants of health

Socioeconomic inequalities in the exposure to environmental risk factors indicate that these risk factors were significantly concentrated among the poor in all the countries (Table 4). This gradient implied that poorer households were more exposed to unimproved drinking-water sources, unsanitary toilets and unclean cooking sources, when compared to the better-off households in all the countries. The magnitude of the ‘pro-poor’ socioeconomic inequality in unimproved sources of drinking water varied substantially between countries and was highest in Zambia (*E*_*c*_ = − 0.3053; *p* < 0.01) followed by Malawi (*E*_*c*_ = − 0.1654; *p* < 0.01). The lowest level was in South Africa (*E*_*c*_ = − 0.0325; *p* < 0.01). Compared to the other SADC countries, as shown in Table 4, Mauritius had lower levels of socioeconomic inequality in both the use of unimproved sanitation facility (*E*_*c*_ = − 0.0016; *p* < 0.01) and unclean cooking fuels/biomass fuels (*E*_*c*_ = − 0.0252; *p* < 0.01). In contrast, Zambia had significantly higher pro-poor inequalities in both unimproved water and unimproved sanitation. Socioeconomic inequality in the use of unclean cooking source was highest in Zimbabwe (*E*_*c*_ = − 0.4297; *p* < 0.01) followed by Swaziland (*E*_*c*_ = − 0.3627; *p* < 0.01), Zambia (*E*_*c*_ = − 0.3203; *p* < 0.01) and South Africa (*E*_*c*_ = − 0.1039; *p* < 0.01).

## Discussion

This paper assesses and compares socioeconomic inequalities in poor SAH and selected SDH across six SADC countries (Malawi, Mauritius, South Africa, Swaziland, Zambia and Zimbabwe). It emerged that socioeconomic inequalities exist in poor SAH among the adult populations in these countries. Except for Mauritius, adults from poorer households report significantly poorer SAH compared to their richer counterparts. Also, socioeconomic inequalities exist in selected NCD risk factors (smoking, excessive alcohol consumption, unhealthy diet and physical inactivity). In all the six SADC countries, poorer households are significantly burdened by adverse environmental risk factors (unimproved drinking water source, unimproved sanitation and unclean cooking energy) compared to their counterparts from richer households.

Studies in both developed [2, 10, 13, 59, 60] and developing countries [9, 17, 61] have found that poor health is disproportionately concentrated among poorer individuals and households compared to their richer counterparts. This ‘pro-poor’ inequality is known in the literature as the “health gradient” [4]. Many factors may contribute to the socioeconomic inequalities in SAH. These inequalities can be partially explained by unequal distribution of SDH (such as education, household wealth, employment, social protection, housing, infrastructure and geographical area of residence) [17, 61]. This implies that factors that lie outside of the health sector have a significant impact on health status and contribute to the observed inequalities in health.

The ‘pro-poor’ pattern of inequality in smoking observed in this paper has been documented in the literature for both developed and developing countries. When stratified by education and income, smoking is more prevalent among people with lower levels of income and educational attainment [18, 42, 62–65]. However, a positive gradient in smoking has been found among women in South Africa, especially among highly educated women [66]. These variations in smoking behaviour in different countries and settings could be attributed to the stages of cigarette epidemic, cultural influences or the effectiveness of domestic tobacco control policies and strategies [62, 66–68]. In recent years smoking rates have declined or even remained relatively constant in many high-income countries. However, they are increasing in many LMICs due to intensified marketing strategies of tobacco companies that aim to attract new smokers especially among the socially disadvantaged groups [18, 68, 69].

Socioeconomic inequality in physical inactivity is mixed. In some studies, a pro-rich pattern exists as individuals from richer households tend to be more physically inactive than those from poorer households [18, 62]. Other studies have reported a pro-poor pattern [42, 70] and this could be more pronounced for women of lower SES than for men [71, 72]. The mixed pattern of inequality in physical inactivity found in this paper may perhaps be explained by contrasting socioeconomic patterns of occupational and leisure-related physical activity across SES groups. A systematic review of evidence concluded that higher SES is positively correlated with leisure-related physical activity, while lower SES is positively associated with occupational physical activity [70]. Unfortunately, the measures used to categorise levels of physical activity in this paper, as in many other papers, do not distinguish between the domains of physical activity at work, during commuting or leisure-time [73]. In many settings, however, disparities in physical activity are often attributable to lack of knowledge of the benefits of physical activity, lack of environments that support physical activity, financial barriers and time constraints [67, 73].

Low levels of physical activity as well as inadequate fruit and vegetable consumption can increase the risk of cardiovascular disease, diabetes, hypertension, cancer and premature mortality [74, 75]. Studies on socioeconomic inequalities in fruit and vegetable consumption (in both developing and developed countries) have shown a pro-rich pattern as the poor (including those with low educational attainment) consume less fruit and vegetables compared to the rich [18, 62, 75, 76]. This pro-rich pattern in fruit and vegetables consumption is also reported for the elderly population in Canada and South Africa [75, 76]. Although other factors need to be examined, an Australian study has shown that people of low SES may have less desire to increase fruit and vegetable intake due to the perceived barriers of price and storage [77].

The generally pro-rich inequalities in heavy episodic alcohol drinking found in this paper are consistent with previous studies which found that excessive alcohol consumption is more concentrated among the better-off [42, 52]. However, some multi-country studies point to inter-country variations. In fact, two distinct patterns of inequality emerge across countries (with both pro-poor and pro-rich inequality) when education and income levels are used as proxy measures of SES [18, 78]. Diverging patterns in socioeconomic inequality in alcohol use could be partly attributed to differences in drinking practices, both between and within countries, as well as variations that exist in the socioeconomic distribution of consumers of different types of alcoholic beverages [79].

This paper also underscores the importance of environmental determinants in health inequalities. Countries with a higher proportion of individuals exposed to unsafe drinking water, unimproved sanitation and unclean cooking energy have a larger proportion of individuals with poorer SAH. The literature supporting this paper’s findings on socioeconomic inequalities in environmental determinants of health is far less convoluted. Indeed, exposure to environmental risk factors is linked to adverse health outcomes [80]. For example exposure to indoor air pollution alone is implicated in about 2 million deaths in developing countries and this accounts for about 4% of the global burden of disease [80].

Considering the observed inequalities in all the NCD risk factors assessed in this paper, studies have shown that many of these ill-health related behaviours are generally associated with poor SAH of respondents [19, 81]. This suggests that when a behaviour is less healthy, self-perceived health is negatively affected [19]. Thus, while this may not be entirely a cause and effect link, it is not surprising that the patterns of inequality in ill-health and inequalities in these NCD risk factors have emerged in this paper. Also, in relation to environmental risk factors considered in this paper, it has been documented elsewhere that these environmental determinants of ill-health, contribute significantly to the disparities in morbidity and mortality [17, 82]. In South Africa, for instance, access to good sanitation, clean source of cooking energy, and access to potable drinking water have a significant impact on disparities in good health [17]. In the Americas, also, lack of access to clean water and sanitation facilities at the household level significantly contributes to disparities in life expectancy, infant mortality and maternal mortality in the region [82]. Similar conclusions have emerged from a systematic review of evidence from European countries [83].

The results in this paper have important implications for policy as the goal of reducing health inequalities needs to be incorporated within the goals of national health systems. Because poor SAH is concentrated among the poor and the SDH are also generally distributed to the disadvantage of the poor, it is very likely that meeting the needs of the disadvantaged populations will improve the overall health status of the population in these countries [3, 17]. This resonates with the Sustainable Development Goals (SDGs) and the context of current health policy debates and the call for national health systems to move towards universal health coverage (UHC) [84, 85]. Because there is a relationship between socioeconomic inequalities in the SDH and socioeconomic inequalities in ill-health, tackling the SDH will substantially lead to reductions in health inequality. There is a need for collaborative efforts and actions from sectors other than health to tackle health inequalities. This is because many of the SDH that need to be addressed lie outside the ambit of the health sector. In fact, there are recent calls to strengthen “intersectoral collaboration” to improve overall health and reduce inequalities in health [86, 87]. So, while this paper does not aim to provide specific policy strategies to address the SDH including the risk factors of NCDs in each SADC country, it has highlighted the importance of significant domains outside the direct control of the health sector that require multi-sectoral action. This evidence can stimulate the ongoing policy debate among scholars and policy makers to identify effective pathways to enhance inter alia “intersectoral action for health” to address the determinants of health and to improve health outcomes and reduce disparities in health [86, 87].

Although similar patterns of inequalities have been reported for many of the conditions considered in this paper, and the need to address the SDH have been noted, there is not a specific magic bullet for tackling these disparities that can be applied equally across all the SADC countries. Thus, it is important for policy interventions in individual SADC countries to take cognizance of peculiarities within the country. And to do this with an overall aim of ensuring that comprehensive and carefully tailored country-specific policy interventions are put in place that address the needs of the poor and socially disadvantaged population groups. Further, and importantly, these policies need to be monitored with an equity lens.

One of the strengths of the paper is the use of the WHS dataset that is based on the same set of questionnaires and methodology. It provides a good basis for cross-country analysis and comparison. Also, this study provides a comprehensive analysis of both inequalities in ill-health and the SDH including the risk factors of NCDs. This represents an initial attempt for such analysis in the context of the SADC region. Further, this paper focuses on an indicator of general health status (SAH), a multi-dimensional measure, instead of individual disease conditions. However, with SAH, there may be differences in the meaning that individuals accord to their health and illness as well as variations in standards or expectations about what constitutes “good health” between socioeconomic groups [88]. This notwithstanding, SAH has been used in the literature as it is a validated predictor of mortality and morbidity [39, 89]. In addition, SAH and health risk factors were self-reported, which could have introduced recall bias [52]. Self-reports of health risk behaviours are more prone to either under-reporting or over-reporting in health surveys because of social desirability bias. This is associated with the tendency of participants to answer survey questions in a socially desirable manner, as a strategy for conforming to social norms and gaining social acceptance. The self-report patterns may be different between countries and SES groups. However, the findings of this paper are in line with international literature regarding the so-called gradient in health and risk factors [4]. In addition, dichotomising responses of SAH (i.e. good and poor health) might have resulted in the loss of information, which in turn, may have some effect on reported inequality. Even though this effect is hard to measure, this dichotomisation has been used in similar studies [17]. Another limitation relates to the WHS data that were collected over a decade ago. This dataset remains invaluable for cross-country comparison as the same set of questionnaires has been used. In fact, the WHS data have been used in recent studies [90, 91].

For future studies, it is suggested that comparable nationally representative datasets may be used to assess patterns and trends in inequality, and the impact of economic and structural changes on the patterns of inequality in the SADC over time. Also, while it is important to estimate and to assess the nature of inequalities in health and the SDH, there is a need for studies that go beyond quantifying inequality, to investigate the underlying drivers, in terms of factors or determinants of health, that influence the distribution of such inequalities in the SADC context and an assessment of policy initiatives within countries to tackle disparities in ill-health.

## Conclusion

Good health is indispensable for general well-being and economic growth. The results in the paper show the existence of socioeconomic inequalities in SAH, NCDs risk factors and environmental risk factors across the six SADC countries. In many cases, these inequalities are to the disadvantage of the poor. To a great extent, and based on previous research, it can be argued that inequalities in these determinants of health explain inequalities in poor SAH in the SADC region. Thus, tackling inequalities in these determinants of health, which require inter-sectoral action, would substantially contribute to health improvements and to reductions in health inequality both within and between countries.
